# An Oligoimide Particle as a Pickering Emulsion Stabilizer

**DOI:** 10.3390/polym10101071

**Published:** 2018-09-27

**Authors:** Yu-Jin Cho, Dong-Min Kim, In-Ho Song, Ju-Young Choi, Seung-Won Jin, Beom-Jun Kim, Jin-Won Jeong, Chae-Eun Jang, Kunmo Chu, Chan-Moon Chung

**Affiliations:** 1 Department of Chemistry, Yonsei University, Wonju 26493, Korea; chyujn904@naver.com (Y.-J.C.); dmkimr@yonsei.ac.kr (D.-M.K.); segunda@nate.com (I.-H.S.); cjy0510@yonsei.ac.kr (J.-Y.C.); jinsw0906@yonsei.ac.kr (S.-W.J.); sk754@naver.com (B.-J.K.); jjw9172@naver.com (J.-W.J.); minnie0511@naver.com (C.-E.J.); 2 Samsung Advanced Institute of Technology, Suwon 16678, Korea; chukunmo@gmail.com

**Keywords:** Pickering emulsion, stabilizer, oligoimide particle, microencapsulation

## Abstract

A pyromellitic dianhydride (PMDA) and 4,4′-oxydianiline (ODA)-based oligoimide (PMDA-ODA) was synthesized by a one-step procedure using water as a solvent. The PMDA-ODA particles showed excellent partial wetting properties and were stably dispersed in both water and oil phases. A stable dispersion was not obtained with comparison PMDA-ODA particles that were synthesized by a conventional two-step method using an organic solvent. Both oil-in-water and water-in-oil Pickering emulsions were prepared using the oligoimide particles synthesized in water, and the size of the emulsion droplet was controlled based on the oligoimide particle concentration. The oligoimide particles were tested to prepare Pickering emulsions using various kinds of oils. The oil-in-water Pickering emulsions were successfully applied to prepare microcapsules of the emulsion droplets. Our new Pickering emulsion stabilizer has the advantages of easy synthesis, no need for surface modification, and the capability of stabilizing both oil-in-water and water-in-oil emulsions.

## 1. Introduction

A Pickering emulsion is an emulsion stabilized by a colloidal suspension of finely divided solids [1,2,3,4,5]. The stabilization by solid particles provides specific properties to such emulsions: the high resistance to coalescence is a major benefit. The control of Pickering emulsion stability and type plays an important part in various significant processes, including oil extraction and recovery, emulsion polymerization, and heterogeneous catalysis [2].

For the adsorption of solid particles at the oil–water interface, partial wetting of the solid by both water and oil is required. Some solid particles have been used as prepared without any modification to prepare Pickering emulsions, including silica [6,7], layered double hydroxide [8], hematite [9], Fe_2_O_3_ [10], graphene oxide [11,12], cellulose nanocrystal [13], and polymer particles [14,15]. Generally, the unmodified particles with a specific surface wettability can only form one type of emulsion (oil-in-water or water-in-oil) [2]. To improve the partial wetting properties of the unmodified particles and consequently control the stability and type of Pickering emulsions, modification of the solid surface or pH adjustment of the dispersion medium is extensively performed [1,2,3,4,5]. There are several methods for surface modification, which include either chemical grafting of organic molecules [16,17,18], or the physical adsorption of different types of molecules [19,20,21,22]. The surface modification often requires complex and tedious procedures. Therefore, it is desirable to develop a Pickering stabilizer that is easy to prepare without surface modification and that can stabilize both oil-in-water and water-in-oil type Pickering emulsion systems.

Polyimides are high performance polymers with high thermal stability, good mechanical properties, and excellent chemical resistance, and are widely used in a variety of industries [23,24]. Oligoimides have also been extensively studied, utilizing the advantages of oligomeric polyimide. For example, the relatively low melt viscosity of oligoimides can facilitate the dispersion of inorganic particles in the resulting nanocomposites [25]. Oligoimides form oriented Langmuir-Blodgett films due to their amphiphilicity and controlled short molecular length [26]. In order to improve the poor solubility and processability of polyimides, telechelic oligoimides have been developed [27,28,29]. In this work, oligoimide particles were used for the first time as a stabilizer for the preparation of Pickering emulsions. Oligoimide particles synthesized in aqueous medium have partial wetting properties in both water and oil phases. The stability of the prepared Pickering emulsions was evaluated, and the emulsions were used in microencapsulation applications.

## 2. Materials and Methods 

### 2.1. Materials

Pyromellitic dianhydride (PMDA, 97%), 4,4′-oxydianiline (ODA, 97%), 1-methyl-2-pyrrolidone (NMP, 99%), *n*-hexadecane (99%), pyridine (99%), acetic anhydride (Ac_2_O, 99%), and decahydronaphthalene (mixture of *cis* and *trans* forms, decalin, 99%) were purchased from Sigma-Aldrich (St. Louis, MO, USA). All water was double-distilled. Methyl methacrylate (MMA, 99%) and divinylbenzene (DVB, 80%) were also purchased from Sigma-Aldrich and used as shell materials. 2,2′-Azobis(2-methylpropionitrile) (AIBN, 98%) was purchased from Sigma-Aldrich and was used as a radical initiator for the polymerization of MMA and DVB. Sulfuric acid (95%~98%) was purchased from Sigma-Aldrich and was used for viscosity measurements. Acetone (>99%) and methanol (>99%) were purchased from SK Chemicals (Seongnam, Korea).

### 2.2. Instruments

An ultrasonicator (VCX750, Sonics & Materials, Newtown, CT, USA) was used to disperse the oligoimide particles in water (or an oil) with a power of 150 W and a frequency of 30 kHz. The polyimide particles synthesized in NMP were ground using a planetary ball mill (Pulverisette 7, FRITSCH, Markt Einersheim, Germany) at 800 rpm for 22 h. Two weight-balanced grinding cups were placed in the mount, with each cup containing nine zirconium oxide grinding balls (1 mm in diameter) and polyimide particles. A homogenizer (T18, IKA, Staufen, Germany) was used to prepare emulsions.

### 2.3. Characterization

IR spectra were recorded on a Fourier transform infrared (FT-IR) spectrophotometer (Spectrum One B, Perkin Elmer, Waltham, MA, USA). Elemental analysis (EA) was carried out by using a Flash 2000 CHNS/O analyzer. A fluorescence microscope (BX-51, Olympus, Tokyo, Japan) equipped with X20 objective lenses with a numerical aperture of 0.46 was used to take pictures of the emulsions. The sizes of the emulsion droplets were analyzed using a CCD camera (HK6U3Cool, KOPTIC, Seoul, Korea) equipped in the microscope and image analysis software (HKBasic, KOPTIC). The mean diameter and size distribution were determined from a data set of at least 1000 measurements for each sample. The measurement of contact angles of the water drop on pressed oligoimide tablets in air was performed at room temperature by the sessile drop method using a Phoenix 300 contact angle goniometer (SEO Co, Suwon, Korea) [30]. The volume of the water drop was 6 μL. The pressed tablets of an oligoimide sample were prepared by compacting the particles under a pressure of 9 metric tons applied by a KBr press (KBr Manual Hydraulic Press, Perkin Elmer, Waltham, MA, USA). All the measurements were conducted in triplicate. A field emission scanning electron microscope (FE-SEM) (SU-70, Hitachi, Tokyo, Japan) was used to examine PMDA-ODA particles and the microcapsules at an accelerating voltage of 30 kV. Thermogravimetric analysis (TGA) of oligoimide particles and microcapsules was carried out on a TGA instrument (TGA-50, Shimadzu, Kyoto, Japan) under a nitrogen flow of 50 mL/min at a heating rate of 10 °C/min. Differential scanning calorimetry (DSC) was conducted under a nitrogen flow rate of 50 mL/min and a heating rate of 30 °C/min. For the first run in DSC and in TGA, the samples were heated to 300 °C. After cooling to room temperature, a second run was performed. The conductivity values of dispersions and emulsions were measured using a digital conductivity meter (4365 Traceable^®^ Conductivity Pen, Control Company, Webster, TX, USA). Dynamic light scattering (DLS) (DLS-7000, Otsuka Electronics, Osaka, Japan) was used to measure the distribution of particle sizes. The DLS study of aqueous oligoimide particle dispersion (0.01 wt %) was carried out at a scattering angle of 115° at 25 °C. Inherent viscosities were determined using a viscometer (Cannon-Fenske type, Sigma-Aldrich, St. Louis, MO, USA) at a concentration of 0.5 g/dL in concentrated sulfuric acid at 30 °C.

### 2.4. Preparation of PMDA-ODA Particles in Water

PMDA (1.09 g, 5.00 mmol) and distilled water (19 mL) were put into a dried 100-mL two-neck round-bottom flask fitted with a condenser and a nitrogen gas inlet. The flask was heated in an oil bath and the dispersion became a homogeneous solution. After the flask was heated to 100 °C, ODA (1.00 g, 5.00 mmol), pyridine (0.800 mL, 10.0 mmol), and acetic anhydride (0.940 mL, 10.0 mmol) were added. The resultant mixture was stirred under nitrogen atmosphere for 24 h at 100 °C. After cooling to room temperature, the reaction mixture was poured into a mixed solvent of distilled water and methanol. A solid was collected by filtration. Washing with water (100 mL), methanol (100 mL), and acetone followed by drying in a vacuum afforded a yellowish powder of PMDA-ODA. The obtained powder was ground in a mortar for 10 min before use. FT-IR (KBr, cm^−1^) PMDA-ODA: 1778 (imide C=O asymmetric stretch), 1728 (imide C=O symmetric stretch), and 1378 (imide C–N stretch). Elemental analysis calcd. C: 69.11, H: 2.64, N: 7.33%; Found C: 68.73, H: 2.87, N: 7.65%. Inherent viscosity: 0.15 dL/g.

### 2.5. Preparation of PMDA-ODA Particles in NMP

PMDA (1.09 g, 5.00 mmol) and ODA (1.00 g, 5.00 mmol) in NMP (19 mL) were put into a dried 100-mL two-neck round-bottom flask fitted with a condenser under nitrogen atmosphere. The resultant solution was stirred in an ice bath for 2 h and then at room temperature for 22 h, yielding a clear and viscous solution of poly(amic acid) [23,24]. The poly(amic acid) was imidized by the addition of acetic anhydride (0.940 mL, 10.0 mmol) and pyridine (0.800 mL, 10.0 mmol), and subsequent heating at 160 °C for 6 h. The resultant polyimide-containing mixture was cooled to room temperature and then poured into distilled water. The resulting precipitate was collected by filtration. Washing with water (100 mL), methanol (100 mL), and acetone followed by drying in a vacuum afforded a yellowish powder of PMDA-ODA. The obtained powder was ground in a mortar for 10 min before use. FT-IR (KBr, cm^−1^) (Figure S1): PMDA-ODA: 1775 (imide C=O asymmetric stretch), 1722 (imide C=O symmetric stretch), and 1381 (imide C–N stretch). Inherent viscosity: 0.35 dL/g. 

### 2.6. Preparation of Reference PMDA-ODA Sample for Degree of Imidization (DI) Measurement 

PMDA-ODA was synthesized using water by the method described above. The reference PMDA-ODA sample was prepared by the following thermal treatment. The temperature was increased stepwise to 100 and 200 °C, and the samples were allowed to stand at each temperature for 1 h. Finally, the imidization was completed by heating at 300 °C for 2 h. 

### 2.7. DI Measurement

The degree of chemical imidization of PMDA-ODA was calculated using Equation (1):

(1) $$\mathrm{DI}\;\left(\%\right)=\frac{A_{s}\left(imide\;C–N\;stretch\right)/A_{s}\left(C=C\;aromatic\;stretch\right)}{A_{R}\left(imide\;C–N\;stretch\right)/A_{R}\left(C=C\;aromatic\;stretch\right)}\times 100$$

where *A_S_* and *A_R_* are the absorbance values of the sample and reference, respectively [31]. Absorption bands of imide C–N stretch at 1378 cm^−1^ and C=C aromatic stretch at 1500 cm^−1^ were used. The aromatic absorption band was used as an internal standard to normalize the variations. The ratio of two absorbance values of a sample PMDA-ODA was compared with that of a reference PMDA-ODA to obtain DI values. The band absorbance ratio of the reference PMDA-ODA prepared by the thermal treatment was taken to be equivalent to that of the completely imidized PMDA-ODA, because the completely imidized PMDA-ODA showed no difference in the band absorbance ratio [31,32,33].

### 2.8. Preparation of Pickering Emulsions Stabilized by PMDA-ODA Particles Synthesized in Water 

Pickering emulsions with different oil-water volume ratios (1:6, 1:1, and 6:1) were prepared. In the cases of Pickering emulsions with a 1:6 ratio, PMDA-ODA particles (0.1, 0.2, 0.5, or 1.0 wt %) were put into water (30 mL) to make a dispersion, which was subjected to ultrasonication for 30 min. An oil-in-water Pickering emulsion was prepared by adding 5 mL of an oil (*n*-hexadecane, linseed oil, olive oil, or phenyl acetate) to the dispersion and agitating at 20,000 rpm with a homogenizer for 5 min. In the case of the emulsions with an oil-water volume ratio of 6:1, oligoimide particles (0.5 wt %) were put into decalin or phenyl acetate (30 mL) to make a dispersion, which was then subjected to ultrasonication for 30 min. Water-in-oil Pickering emulsions were prepared by adding 5 mL of water to the dispersions and homogenizing at 20,000 rpm for 30 s. Pickering emulsions with a 1:1 oil-water volume ratio were also prepared using decalin (15 mL) and water (15 mL). Firstly, oligoimide particles (1.0 wt %) were ultrasonically dispersed into decalin, and then water was added to the resultant dispersion. Secondly, oligoimide particles (1.0 wt %) were ultrasonically dispersed into water, and then decalin was added to the resultant dispersion. In both cases, homogenizing was conducted at 20,000 rpm for 5 min. In all cases described above, photographs and micrographs of the resultant emulsions were taken 48 h after emulsification.

### 2.9. Conductivity Measurements 

The conductivities of the PMDA-ODA particle dispersions and Pickering emulsions were measured immediately after preparation of the dispersions and emulsions at room temperature. The measurements were conducted with gentle stirring to avoid creaming. 

### 2.10. Drop Test

One drop of each emulsion was added to both water and oil (*n*-hexadecane, linseed oil, olive oil, decalin, or phenyl acetate), and its ease of dispersion was assessed by visual inspection. Relatively rapid dispersion indicated that the continuous phase of the emulsion was the same as that of the diluent [34].

### 2.11. Percentage of Survived Emulsion Measurements 

The percentage of surviving emulsion was calculated using Equation (2):

(2) $$\mathrm{Emulsion}\;\mathrm{remaining}\;\left(\%\right)=100-\left(\frac{V_{separated\;oil}}{V_{initial\;oil}}\times 100\right)$$

where *V_initial oil_* is the initial oil volume prior to emulsification, and *V_separated_*_*oil*_ is the volume of separated oil [34].

### 2.12. Microencapsulation 

To a 0.5 wt % PMDA-ODA particle dispersion in water (30 mL), an oil phase containing 3.200 g *n*-hexadecane, 0.400 g MMA, 0.400 g DVB, and 0.010 g AIBN was added to a 50 mL vial. A stable Pickering emulsion was generated by homogenizing at 20,000 rpm for 5 min. Then, the obtained emulsion was heated at 68 °C for 16 h under nitrogen atmosphere to induce radical polymerization inside the emulsion droplets. The obtained microcapsules were filtered, washed with water and ethyl alcohol, and dried under ambient conditions.

## 3. Results and Discussion

### 3.1. Synthesis and Properties of Oligoimides

**Scheme 1:**
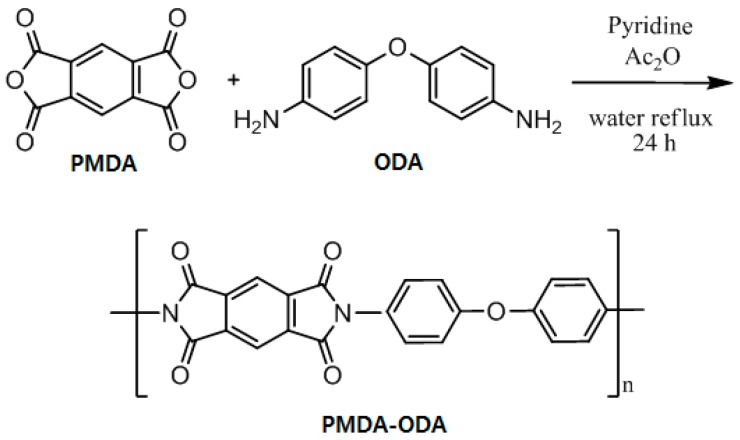
Synthesis of PMDA-ODA oligoimide in water.

**Figure 1:**
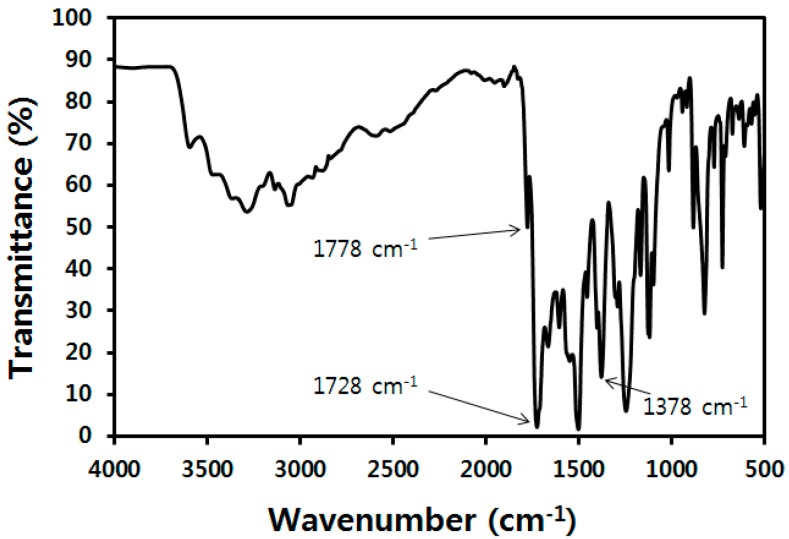
FT-IR spectrum of PMDA-ODA oligoimide synthesized in water.

The PMDA-ODA was synthesized in a powder form by a one-step procedure (Scheme 1 and Table 1). It should be noted that water was used as a reaction solvent, which is in contrast to the conventional synthesis method of polyimides using toxic organic solvents, such as NMP and *m*-cresol [31,35,36]. The formation of the PMDA-ODA was confirmed by FT-IR spectroscopy (Figure 1) and elemental analysis. The absorption bands of the imide ring were observed at 1778 and 1728 cm^−1^ (due to imide C=O asymmetric and symmetric stretch, respectively) and 1378 cm^−1^ (due to imide C–N stretch) [31,36]. The elemental analysis values of PMDA-ODA were in good accordance with the calculated values. A comparison PMDA-ODA was also prepared using NMP by a conventional two-step method and characterized by FT-IR spectroscopy (Figure S1).

Table 1 lists the synthetic yield, viscosity, DI, and thermal properties of the PMDA-ODA synthesized in water. The yield and DI of the PMDA-ODA were 97% and 95%, respectively. Because PMDA-ODA was insoluble in organic solvents, its inherent viscosity was measured in concentrated sulfuric acid. Because the viscosity value is relatively low (0.15 dL/g), the PMDA-ODA is considered to be an oligoimide. A higher viscosity polyimide was not obtained in the reaction system using water as the solvent. Thermal decomposition and glass transition temperatures of PMDA-ODA were studied by TGA and DSC, respectively, under nitrogen atmosphere. The thermal properties are lower than those of polyimides synthesized by a conventional two-step method using organic solvents [37], but are much higher than those of most other polymers [38].

### 3.2. Characterization and Dispersion of Oligoimide Particles

**Figure 2:**
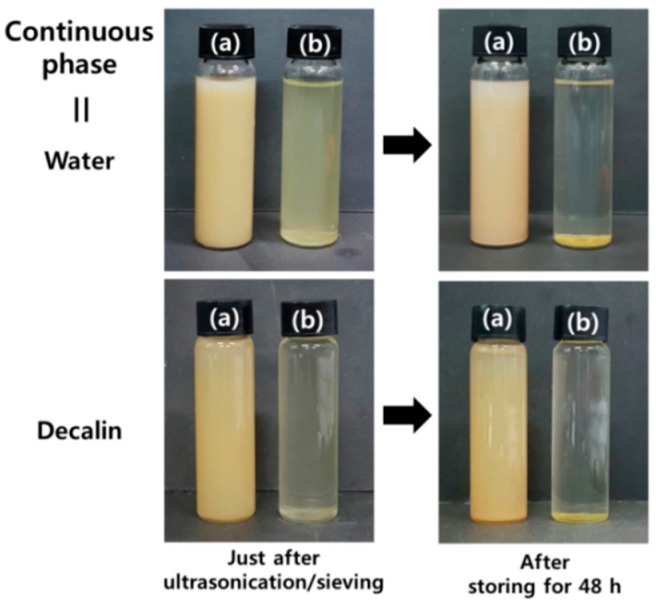
Photographs of dispersions of PMDA-ODA particles in water (**top**) and decalin (**bottom**) synthesized using (**a**) water and (**b**) NMP. The photographs were taken just after ultrasonication/sieving and after storing the samples for 48 h at room temperature.

The PMDA-ODA particles were dispersed into water or decalin, and the stabilities of the resulting dispersions were evaluated (Figure 2). For comparison, PMDA-ODA particles prepared in NMP were also tested. After the particles were ground and sieved with a 200 µm aperture sieve, they were dispersed in distilled water or decalin. Sieving was conducted to remove very large particles in order to rule out the possible effect of them on dispersion. After ultrasonication at a frequency of 30 kHz for 30 min, the dispersions were sieved again with a 25 µm aperture sieve. The dispersions just after ultrasonication and sieving are shown in Figure 2 (left). The amount of particles that passed through the sieves and that were uniformly dispersed was much greater in the case of the particles synthesized in water. After storing the samples for 48 h, the particles synthesized in water were still stably dispersed, but the particles synthesized in NMP settled at the bottom of the vial (Figure 2, right). This indicates that the oligoimide particles synthesized in water have a good dispersion stability in both water and decalin, but the particles synthesized in NMP do not. 

Most PMDA-ODA particles prepared in NMP were greater than 30 µm in size after ultrasonication (Figure S2a). Because it was thought that the poor dispersion of the particles synthesized in NMP might be due to their large size, ball milling was conducted to prepare finer particles using a planetary ball mill with a speed of 800 rpm for 22 h. The sizes of resultant particles were greater than 5 µm, as shown in Figure S2b. The ball-milled particles were dispersed in water or decalin by homogenizing them, but a stable dispersion could not be obtained. The incapability of the particles prepared in NMP to form a stable dispersion may be attributed to their size being bigger than colloidal size. Therefore, the following experiments were conducted using the PMDA-ODA particles synthesized in water.

**Figure 3:**
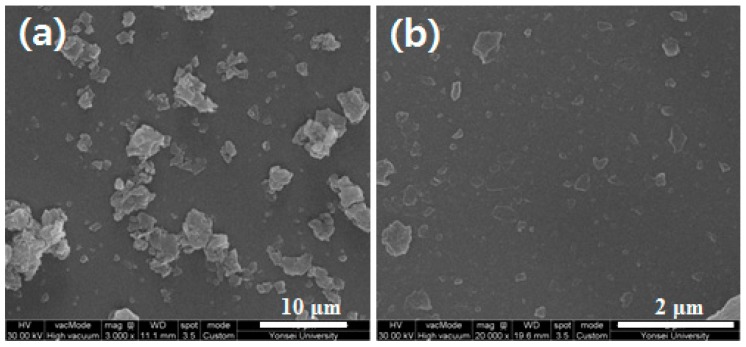
FE-SEM images of PMDA-ODA particles synthesized in water (**a**) before ultrasonication and (**b**) after ultrasonication for 30 min.

**Figure 4:**
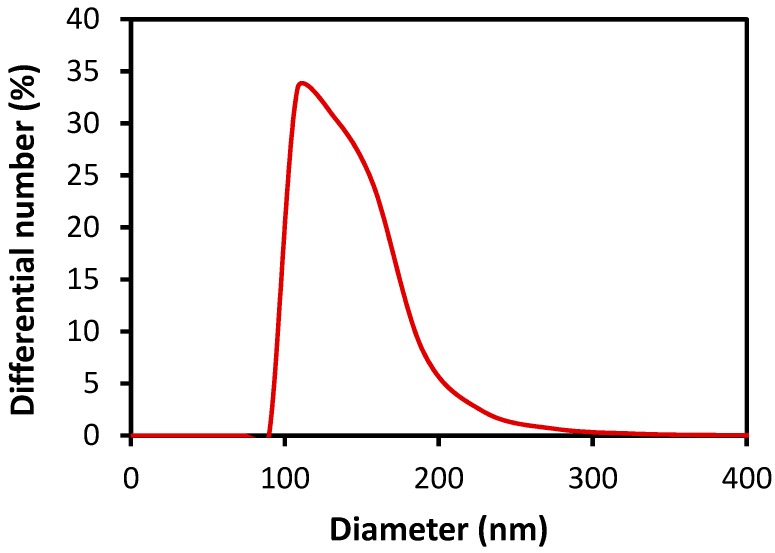
Size distribution of PMDA-ODA particles dispersed in distilled water (0.05 wt %) measured by dynamic light scattering (DLS).

As shown in Figure 3a, the pristine PMDA-ODA particles synthesized in water had a size range of 1–5 µm. When they were put into water, a homogeneous dispersion could not be obtained. Ultrasonication of the dispersion effectively broke the particles into much smaller 100–300 nm sized particles (Figure 3b), which were stably dispersed in water and decalin as described above. It was confirmed that FT-IR spectra of the oligoimide particles before and after ultrasonication were practically identical, indicating that any chemical change did not occur upon sonication. It was found that the ultrasonicated particles had a plate-like shape (Figure 3b). Among reported Pickering emulsion stabilizers, clay and layered double hydroxide (LDH) particles have a plate-like shape and produce stable emulsions [8,39,40,41,42]. The size distribution of the PMDA-ODA particles was investigated using dynamic light scattering (DLS) (Figure 4). The mean size of PMDA-ODA particles was 221 nm (polydispersity index (PDI) = 0.226). The DLS result would be a rough measure of the lateral size distribution of the PMDA-ODA particles because DLS analysis assumes spherical shapes [43,44]. It is considered that the measured value is not the average platelet diameter, but rather the effective hydrodynamic diameter of equivalent spheres described by the tumbling of the platelets [43]. 

The contact angle of water on the PMDA-ODA synthesized in water was measured using a pressed tablet of the oligoimide particles [30]. The measured contact angle (54°) was smaller than 90°, indicating that the PMDA-ODA particles are hydrophilic. This may be mainly attributed to hydrophilic groups on the surface of the PMDA-ODA particles: carboxyl and amino end groups of PMDA-ODA chains and carboxyl and amido groups of incompletely imidized PMDA-ODA repeating units. It is considered that the carboxyl and amino end groups contribute importantly to the hydrophilicity of the particles because PMDA-ODA is oligomeric.

Aqueous dispersions were prepared by varying the wt % of PMDA-ODA particles that were synthesized in water (Table 2). Sieving was not conducted because the particles were fine enough after ultrasonication (Figure 3b and Figure 4). The pH of the dispersions decreased with increasing wt % of the particles. This can be attributed to carboxyl groups on the surface of the particles because the oligoimide chains have carboxyl and amino end groups. More carboxyl groups than amino groups might exist on the particle surface because two carboxyl groups are generated when one anhydride group is hydrolyzed. In addition, amic acid carboxyl groups can remain on the particle surface because the DI of PMDA-ODA is lower than 100%. As expected, the conductivity values of the dispersions increased with decreasing pH.

### 3.3. Oil-in-Water Pickering Emulsions

**Figure 5:**
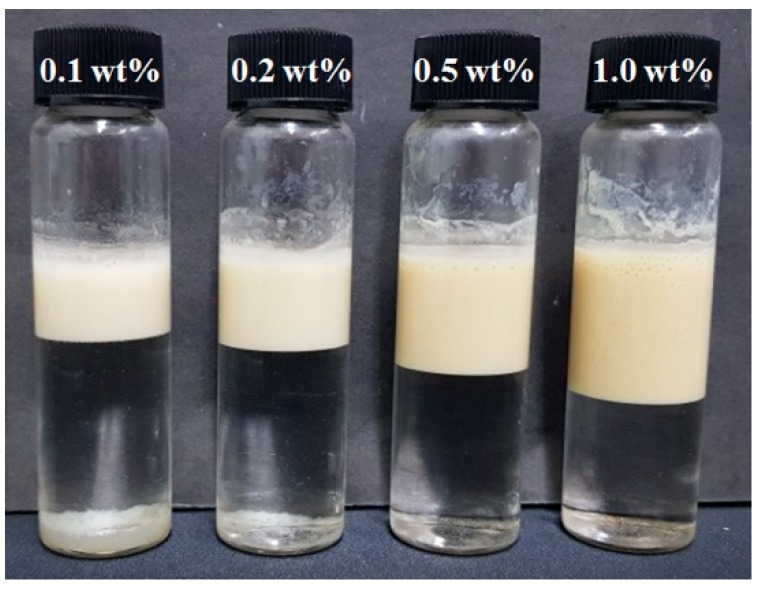
A photograph of PMDA-ODA particle-stabilized *n*-hexadecane-in-water Pickering emulsions prepared at different particle concentrations (0.1–1.0 wt %).

**Figure 6:**
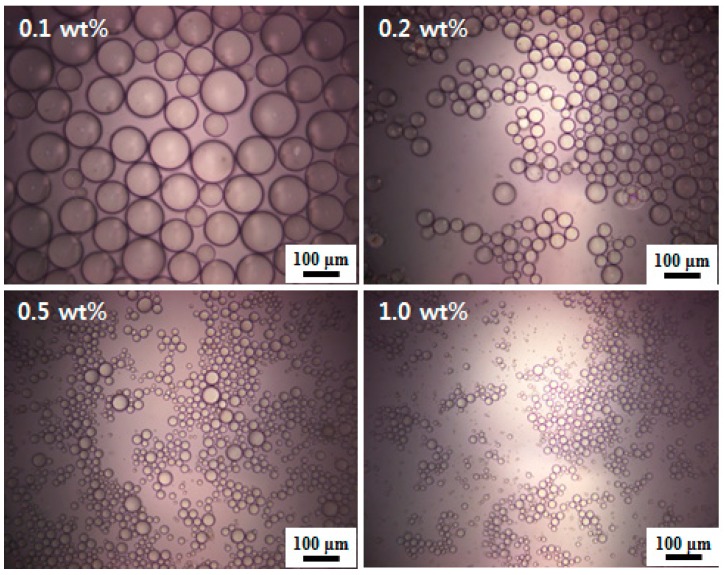
Optical micrographs of PMDA-ODA particle-stabilized *n*-hexadecane-in-water Pickering emulsions prepared at different particle concentrations (0.1–1.0 wt %).

Oil-in-water Pickering emulsions were prepared using *n*-hexadecane as an oil phase and oligoimide particles as a stabilizer (Table 2, Figure 5 and Figure 6). The emulsions were obtained by adding *n*-hexadecane into the aqueous particle dispersions (oil-water volume ratio = 1:6) with different particle wt %. All emulsions obtained were confirmed to be oil-in-water type based on conductivity values higher than 1 µS cm^−1^ [34,45]. This was also supported by drop tests. The percentage of emulsion remaining for 48 h was calculated based on Equation (2), and we found that almost all emulsion droplets survived because no oil-water separation was observed. This result indicates that the Pickering emulsions were completely stable for 48 h after emulsification in the PMDA-ODA particle concentration range from 1.0 to 0.1 wt %. The oil-in-water Pickering emulsions that were stored for 48 h are shown in Figure 5. Although slow creaming was observed with time, the emulsions returned to their original homogeneously dispersed state by light shaking. With increasing storing time, the emulsion layer heights gradually reduced. For example, in the case of the 1.0 wt % particle concentration emulsion, the emulsion layer height reduced to about a half of the original height three months after preparation. It was later found that the Pickering emulsions were stable for six months without phase separation. The mean oil droplet diameters were measured and ranged from 11 to 118 µm. There is a clear trend of decreasing oil-in-water emulsion droplet size with increasing particle concentration, which can be observed in Table 2 and Figure 6. This can be explained by the smaller emulsion droplets having a larger surface area that can be stabilized by a larger amount of oligoimide particles. The size distribution of the oil droplets is shown in Figure S3. In addition to the 1:6 oil-water volume ratio, the 1:1 decalin-water volume ratio was also tested and oil-in-water Pickering emulsions were obtained (particle concentration = 1.0 wt %) (data not shown). 

It has been reported that the stability of Pickering emulsions based on conventional solid particles can often be influenced by pH [46,47,48]. In this case, pH adjustment of the dispersion medium is generally required to prepare stable emulsions. In the present study, it should be noted that stable emulsions were prepared using the oligoimide particles without pH adjustment. 

**Figure 7:**
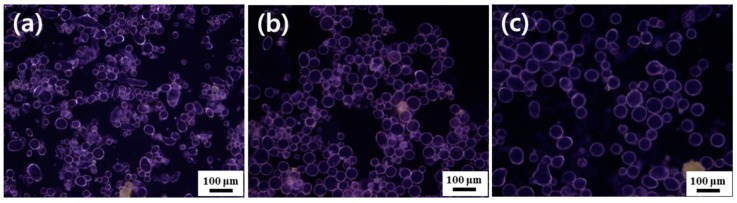
Fluorescence micrographs of oil-in-water Pickering emulsions of three different oils stabilized by PMDA-ODA particles (0.5 wt %): (**a**) linseed oil, (**b**) olive oil, and (**c**) phenyl acetate. Fluorescence micrographs are provided for more clarity (excitation wavelength = 330~380 nm).

To investigate the applicability of the oligoimide stabilizer to various oils, *n*-hexadecane, linseed oil, olive oil, and phenyl acetate were used as oil phases to prepare Pickering emulsions at a particle concentration of 0.5 wt % (Table 3) The pH and conductivity of the aqueous particle dispersion were 4.10 and 243 μS cm^−1^, respectively. All emulsions obtained were confirmed to be oil-in-water type based on the drop test results and conductivity values higher than 1 μS cm^−1^. The droplet size of the emulsions ranged from 19 to 59 µm. Figure 7 shows fluorescence micrographs of oil-in-water Pickering emulsions of three different oils (linseed oil, olive oil, and phenyl acetate) stabilized by PMDA-ODA particles. The emulsion droplets can be seen by fluorescence microscopy because PMDA-ODA shows a weak fluorescence property [49]. In this study, all oil-in-water Pickering emulsion droplets were stable in the aqueous continuous phase, and nearly 100% of them survived for 48 h. It was later found that they were stable for over six months without phase separation. These results indicate that PMDA-ODA particles can stabilize Pickering emulsions of various oils.

### 3.4. Water-in-Oil Pickering Emulsions

**Figure 8:**
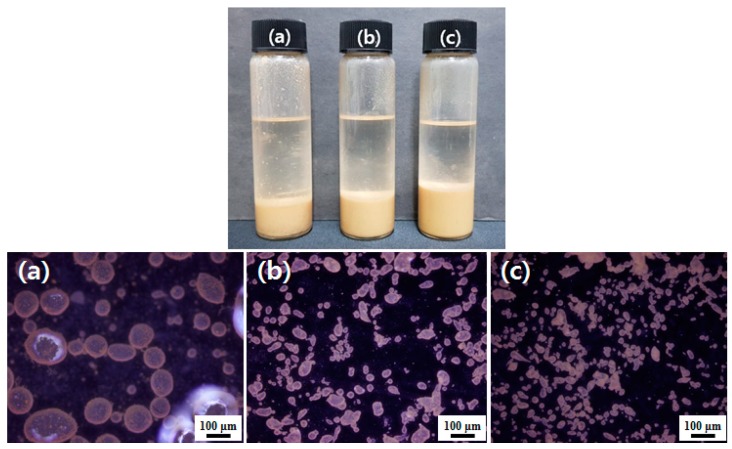
A photograph (**top**) and fluorescence micrographs (**bottom**) of water-in-decalin Pickering emulsions prepared at different PMDA-ODA particle concentrations: (**a**) 0.5 wt %, (**b**) 1.0 wt %, and (**c**) 1.5 wt %. The volume ratio of oil-water was 6:1. Fluorescence micrographs are provided for more clarity (excitation wavelength = 330~380 nm).

PMDA-ODA particles also stabilized water-in-oil Pickering emulsions. Water-in-decalin and water-in-phenyl acetate emulsions were prepared with a 6:1 volume ratio of oil-water and are shown in Figure 8 and Figure S4, respectively. The formation of water-in-oil emulsions was confirmed by the drop test. A conductivity measurement could not be conducted because organic solvents can damage the conductivity meter. Droplets of the emulsions were spherical at a 0.5 wt % particle concentration, but had somewhat irregular shapes at higher concentrations. The droplet size decreased with increasing particle concentration. We found that the water-in-oil Pickering emulsions were completely stable for six months without phase separation. The applicability of the oligoimide particles to both oil-in-water and water-in-oil Pickering emulsions was attributed to the excellent partial wetting properties and colloidal stability of the particles [50].

### 3.5. Microencapsulation

**Figure 9:**
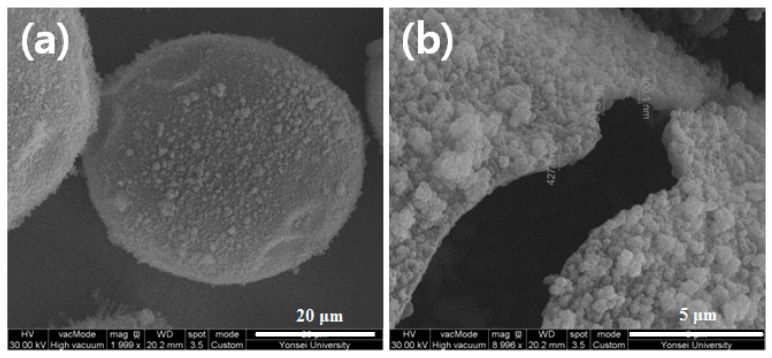
FE-SEM images of (**a**) *n*-hexadecane-loaded microcapsules prepared by oil-in-water Pickering emulsion templating and (**b**) the microcapsule shell wall.

**Figure 10:**
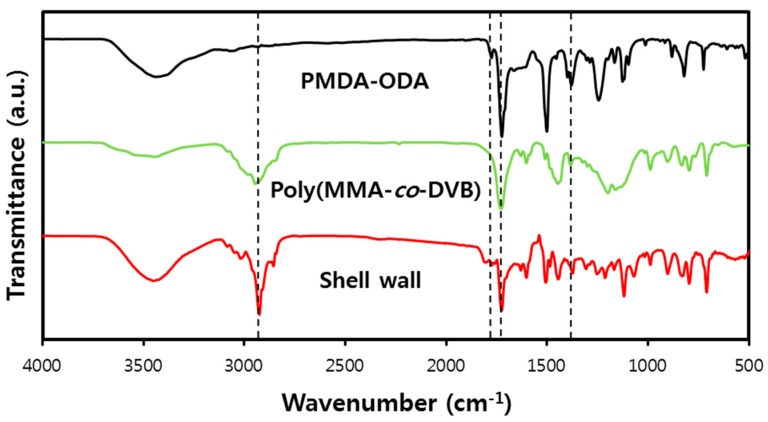
FT-IR spectra of PMDA-ODA, poly(MMA-*co*-DVB), and the shell wall. The shell wall sample was obtained by pressing the microcapsules between two slide glasses and drying.

Microencapsulation of Pickering emulsion droplets was performed as a preliminary evaluation of the applicability of the Pickering emulsion stabilized by oligoimide particles. An oil phase containing *n*-hexadecane, MMA, DVB, and AIBN was added to the PMDA-ODA particle aqueous dispersion (0.5 wt %). A stable oil-in-water Pickering emulsion was obtained by homogenizing the sample at 20,000 rpm for 5 min. The emulsion was heated to induce radical crosslinking polymerization of MMA and DVB inside the droplets [51]. As shown in Figure 9, spherical microcapsules were obtained. By pressing the microcapsules between two slide glasses, the core material flowed out from the capsules. An FT-IR spectrum of the core was in accord with that of *n*-hexadecane (data not shown), indicating that *n*-hexadecane was successfully microencapsulated via Pickering emulsions. FT-IR spectroscopy was conducted to confirm the chemical composition of the shell wall (Figure 10). PMDA-ODA showed absorption bands of the imide ring at 1778, 1728, and 1378 cm^−1^, as described above. Poly(MMA-*co*-DVB) has a CH stretching vibration band at 2840–3140 cm^−1^ and an ester carbonyl stretching band at 1730 cm^−1^. The above absorption bands were all observed in the spectrum of the shell wall, indicating that the shell wall is composed of PMDA-ODA and poly(MMA-*co*-DVB).

**Figure 11:**
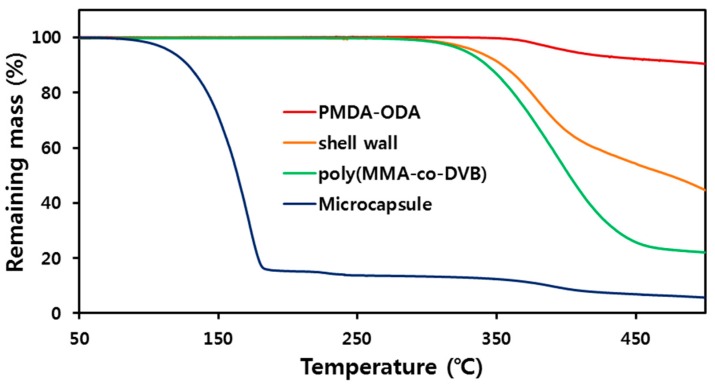
TGA thermograms of PMDA-ODA, the shell wall, poly(MMA-*co*-DVB), and the microcapsule. The shell wall sample was obtained by pressing the microcapsules between two slide glasses and drying.

TGA curves of PMDA-ODA, the shell wall, poly(MMA-*co*-DVB), and microcapsules are shown in Figure 11. Poly(MMA-*co*-DVB) and PMDA-ODA start to degrade around 270 and 350 °C, respectively. T_10_ values for poly(MMA-*co*-DVB) and PMDA-ODA were 343 and 513 °C, respectively. The shell wall showed a T_10_ of 354 °C, which is higher than that of poly(MMA-*co*-DVB). This is attributable to the oligoimide particles contained in the shell wall. The curve for microcapsules is composed of two weight loss steps: the weight loss at 80–180 °C is induced by volatilization of *n*-hexadecane, and the decomposition of shell wall occurs around 370 °C. From the weight loss of *n*-hexadecane, the encapsulation ratio of *n*-hexadecane is calculated to be about 86%. 

## 4. Conclusions

The PMDA-ODA oligoimide particles were synthesized by a one-step procedure using water as a solvent. The PMDA-ODA particles were found to be relatively hydrophilic, and it is considered that the carboxyl and amino end groups contribute importantly to the hydrophilicity of the particles because PMDA-ODA is oligomeric. The PMDA-ODA particles showed partial wetting properties and were stably dispersed in both water and oil phases. In contrast, a stable dispersion could not be prepared from a control sample of PMDA-ODA particles synthesized using an organic solvent (NMP). SEM and DLS showed that the size of the PMDA-ODA particles synthesized in water ranged from 100 to 300 nm after ultrasonication. Both oil-in-water and water-in-oil Pickering emulsions were prepared using the oligoimide particles, and the size of the emulsion droplet was controlled by oligoimide particle concentration. The oil-in-water emulsions were successfully applied to prepare microcapsules of the emulsion droplets. The new oligoimide stabilizer has the advantages of easy synthesis, no need for surface modification, no need for pH adjustment of continuous phase, and the capability of stabilizing both oil-in-water and water-in-oil emulsions.

## Figures and Tables

**Table 1:** Yield, viscosity, degree of imidization (DI), and thermal properties of PMDA-ODA synthesized in water.

Yield (%)	Inherent Viscosity ^a^ (dL/g)	DI ^b^ (%)	Thermal Properties (°C)		
			T_5_^c^	T_10_^d^	T_g_^e^
97	0.15	95	476	513	352

^a^ Measured at a concentration of 0.5 g/dL in concentrated sulfuric acid at 30 °C. ^b^ Degree of imidization calculated from FT-IR spectra using Equation (1). ^c^ Temperature at which a specimen shows 5 wt % decomposition under N_2_. ^d^ Temperature at which a specimen shows 10 wt % decomposition under N_2_. ^e^ Glass transition temperature measured under N_2_.

**Table 2:** Properties of the Pickering emulsions of *n*-hexadecane stabilized by PMDA-ODA particles with different concentrations (oil-water volume ratio = 1:6).

Particle Concentration (wt %)	pH of Dispersion	Conductivity of Dispersion (μS cm^−1^)	Conductivity of Emulsion (μS cm^−1^)	Type of Emulsion Formed ^a^	Emulsion Remaining for 48 h ^b^ (%)	Mean Droplet Diameter ^c^ (μm)
0.1	4.88	60	44	Oil-in-water	~100	~118
0.2	4.47	111	98	Oil-in-water	~100	~45
0.5	4.10	243	201	Oil-in-water	~100	~19
1.0	4.02	287	239	Oil-in-water	~100	~11

^a^ Determined by emulsion conductivity measurements and drop tests. ^b^ Calculated using Equation (2). ^c^ Measured using an optical microscope from a data set of at least 1000 measurements for each sample.

**Table 3:** Properties of the Pickering emulsions of four different oils stabilized by PMDA-ODA particles (0.5 wt %). The volume ratio of oil-water was 1:6.

Oil Used	pH of Dispersion	Conductivity of Dispersion (μS cm^−1^)	Conductivity of Emulsion (μS cm^−1^)	Type of Emulsion Formed ^a^	Emulsion Remaining for 48 h ^b^ (%)	Mean Droplet Diameter ^c^ (μm)
*n*-Hexadecane	4.10	243	201	Oil-in-water	~100	~19
Linseed oil	4.10	243	202	Oil-in-water	~100	~30
Olive oil	4.10	243	200	Oil-in-water	~100	~41
Phenyl acetate	4.10	243	203	Oil-in-water	~100	~59

^a^ Determined by emulsion conductivity measurements and drop tests. ^b^ Calculated by Equation (2). ^c^ Measured using an optical microscope from a data set of at least 1000 measurements for each sample.

## Supplementary Materials

The following are available online at http://www.mdpi.com/2073-4360/10/10/1071/s1. Figure S1: FT-IR spectrum of PMDA-ODA polyimide synthesized in NMP. Figure S2: FE-SEM image of PMDA-ODA particles synthesized in NMP: (a) particles prepared by sieving with a 200 µm sieve and ultrasonication of its dispersion at a frequency of 30 kHz for 30 min; (b) particles prepared by ball milling using a planetary ball mill with a speed of 800 rpm for 22 h. Figure S3: Oil droplet size distributions of PMDA-ODA particle-stabilized *n*-hexadecane-in-water emulsions prepared at various particle concentrations (0.1–1.0 wt %). Figure S4: A photograph and optical micrographs of water-in-phenyl acetate Pickering emulsions prepared with different PMDA-ODA particle concentrations: 0.5, 1.0, and 1.5 wt %.
