# Supplementary material for: An Oligoimide Particle as a Pickering Emulsion Stabilizer
Source: Polymers (Basel). 2018 Sep 27;10(10):1071. doi: 10.3390/polym10101071 (PMC6403722; doi:10.3390/polym10101071)
Supplement: Supplementary file 1 [file polymers-10-01071-s001.pdf]

Supplementary Material

Oligoimide Particle as a Pickering Emulsion Stabilizer

Yu-Jin Cho [1], Dong-Min Kim [1], In-Ho Song [1], Ju-Young Choi [1], Seung-Won Jin [1], Beom-Jun Kim [1], Jin-Won Jeong [1], Chae-Eun Jang [1], Kunmo Chu [2] and Chan-Moon Chung [1,*]

[1] Department of Chemistry, Yonsei University, Wonju 26493, Korea; chyujn904@naver.com (Y.-J.C.); dmkimr@yonsei.ac.kr (D.-M.K.); segunda@nate.com (I.-H.S.); cjy0510@yonsei.ac.kr (J.-Y.C.); jinsw0906@yonsei.ac.kr (S.-W.J.); sk754@naver.com (B.-J.K.); jjw9172@naver.com (J.-W.J.); minnie0511@naver.com (C.-E.J.)

[2] Samsung Advanced Institute of Technology, Suwon 16678, Korea; chukunmo@gmail.com

* Correspondence: cmchung@yonsei.ac.kr; Tel.: +82-33-760-2266

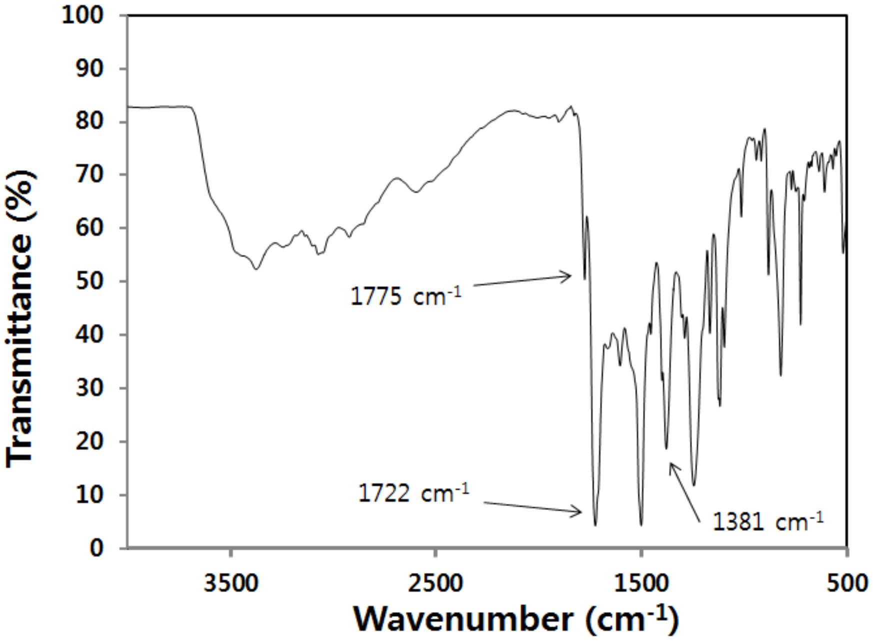

Figure S1. FT-IR spectrum of PMDA-ODA synthesized in NMP.

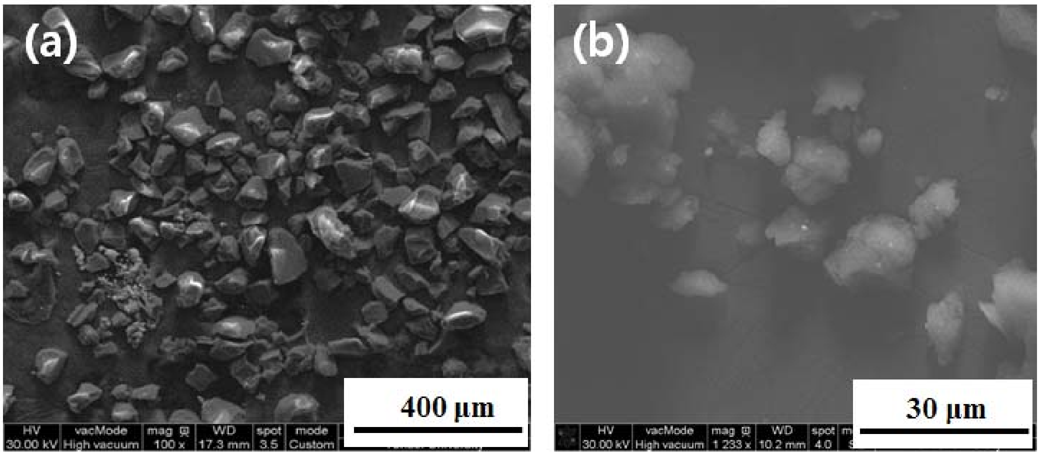

Figure S2. FE-SEM image of PMDA-ODA particles synthesized in NMP: (a) particles prepared by sieving with a 200 µm sieve and ultrasonication of its dispersion at a frequency of 30 kHz for 30 min; (b) particles prepared by ball milling using a planetary ball mill with a speed of 800 rpm for 22 h.

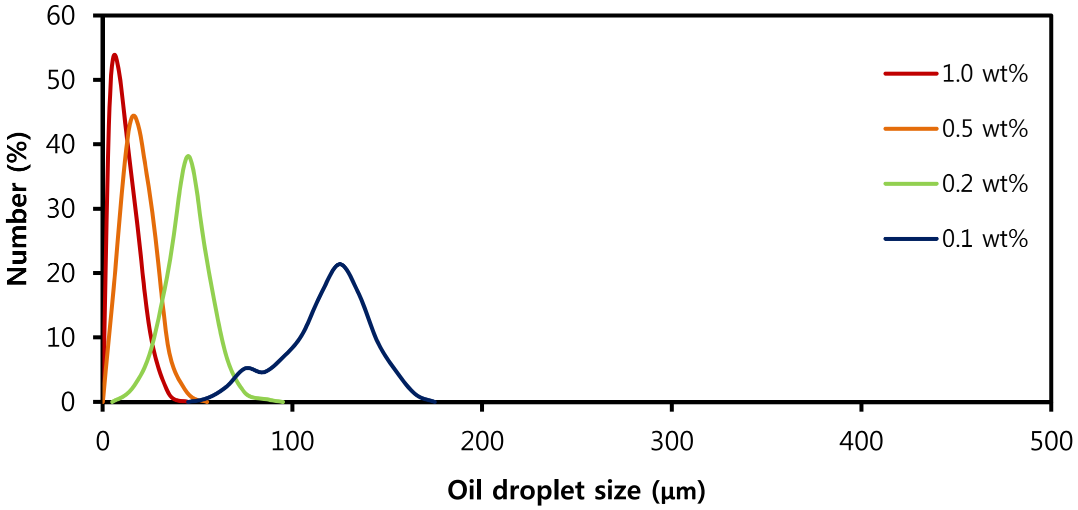

Figure S3. Oil droplet size distributions of PMDA-ODA particle-stabilized *n*-hexadecane-in-water emulsions prepared at various particle concentrations (0.1–1.0 wt %).

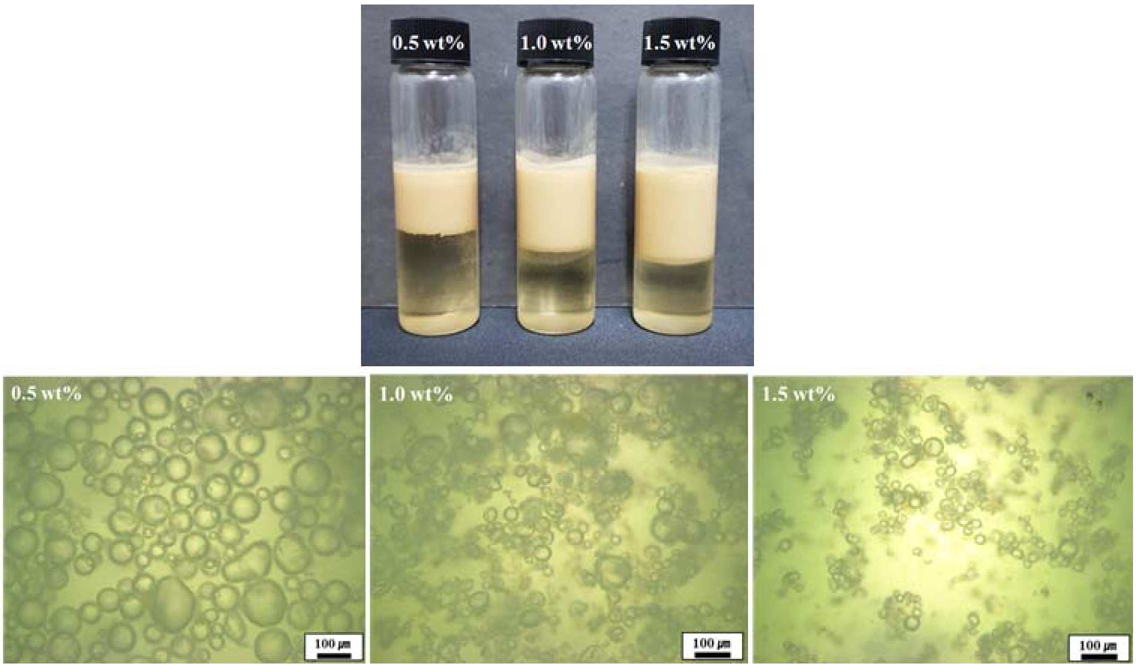

Figure S4. A photograph (top) and optical micrographs (bottom) of water-in-phenyl acetate Pickering emulsions prepared with different PMDA-ODA particle concentrations: 0.5, 1.0 and 1.5 wt %.
